# Calcicole–calcifuge plant strategies limit restoration potential in a regional semi‐arid flora

**DOI:** 10.1002/ece3.7544

**Published:** 2021-05-01

**Authors:** Adam T. Cross, Hans Lambers

**Affiliations:** ^1^ School of Molecular and Life Sciences Curtin University Bentley WA Australia; ^2^ EcoHealth Network Brookline MA USA; ^3^ School of Biological Sciences The University of Western Australia Perth WA Australia

**Keywords:** ecological restoration, edaphic filters, plant development, rehabilitation

## Abstract

**Aim:**

To examine calcicole and calcifuge plant strategies, as well as nutrient‐acquisition strategies, as drivers of the distribution of species in response to edaphic factors, and the degree to which these strategies may act as filters to species establishment in ecological restoration on heavily altered or reconstructed substrates.

**Location:**

An 82,000‐ha area within a major mining province in the Mid‐West region of Western Australia, harboring vegetation communities ranging from species‐poor halophytic scrub on saline flats to dense biodiverse shrubland on the skeletal soils of ancient Banded Ironstone Formations (BIF).

**Methods:**

Univariate and multivariate analyses were employed to examine how variation in soil chemistry and landscape position (undulating plains, slopes, and BIF crests and ridges) influenced patterns of floristic diversity, calcifuge plant strategies, and nutrient‐acquisition strategies in 538 plant species from 830 relevés.

**Results:**

Landscape position was the strongest driver of species richness and vegetation functional composition. Soils became increasingly acidic and P‐impoverished along an increasing elevational gradient. Vegetation from different landscape positions was not compositionally dissimilar, but vegetation of BIF crests and ridges was up to twice as biodiverse as vegetation from adjacent lower‐relief areas and harbored higher proportions of calcifuge species and species with mycorrhizal associations.

**Main conclusions:**

Topographic and edaphic complexity of BIF landforms in an otherwise relatively homogenous landscape has likely facilitated species accumulation over long time periods. They represent musea of regional floristic biodiversity, excluding only species that cannot establish or are inferior competitors in heavily weathered, acidic, skeletal, and nutrient‐impoverished soils. Plant strategies likely represent a major filter in establishing biodiverse, representative vegetation on postmining landforms in geologically ancient regions.

## Graphical abstract

Species assemblages in ancient, heavily weathered landscapes reflect specialized plant–soil relationships. Highly altered substrates typical of postmining landforms offer starkly different environments to native plant communities. Ecological restoration to full ecosystem recovery may not be possible of these landforms within short time periods.

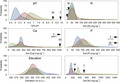

## 1 INTRODUCTION

Calcicole and calcifuge (=acidophilous) plant strategies, which refer to the capacity of species to colonize and persist on calcium‐rich and acidic soils, respectively, influence the distribution patterns of plant species (e.g., Etherington, 1981; Gigon, 1987; Lee, 1999; Ström, 1997; Tyler, 2003; Zohlen & Tyler, 2004). It should be noted that not all calcium‐rich soils are calcareous and alkaline, and hence, calcicole species are not restricted to calcareous soils (Gao et al., 2020). Soil pH is a primary driver of the solubility and plant availability of mineral nutrients, particularly phosphorus (P), iron (Fe), zinc (Zn), and manganese (Mn) that are essential for plant growth (Lambers & Oliveira, 2019; Tyler, 2003). Acidic soils generally exhibit slow nitrification rates (with ${\text{NH}}_{4}^{+}$ a predominant form of plant‐available N) and low availabilities of calcium (Ca), magnesium (Mg), potassium (K), and molybdenum (Mo), as well as greater solubility of aluminum (Al, including potentially toxic forms of Al; Singh et al., 2017; Zheng, 2010), Fe, and Mn (including potentially toxic forms of Mn; Fernando & Lynch, 2015; Lambers et al., 2015; Lambers & Oliveira, 2019; Robson & Loneragan, 1970), and reduced P solubility (Lee, 1999). Calcium‐rich (often calcareous) soils are characterized by rapid rates of nitrification (with ${\text{NO}}_{3}^{‐}$ the predominant form of plant‐available N), as well as low solubility of Fe, Mn, Zn, Cu, and boron (B), and high concentration of Ca^2+^, which may impair plant K uptake (Lee, 1999) or aggravate P sensitivity (Hayes et al., 2019).

Calcicole species are restricted to calcium‐rich soils and typically exhibit insensitivity to Fe and P deficiencies (Lee, 1999; White and Broadley, 2003), and their growth may be inhibited by Al (Clarkson, 1966). Calcifuge species are limited to acidic soils and broadly tolerate high soil concentrations of Al, Mn, and Fe (Clarkson, 1966; Lee, 1999). However, calcifuge species solubilize P and micronutrients such as Fe poorly under calcareous conditions (Zohlen & Tyler, 2000, 2004). They may also exhibit impaired root development when rhizosphere Ca concentrations exceed 1 mM (Jefferies and Willis, 1964; Burstrom, 1968). The relationship between pH and the bioavailability of Ca or Al in natural soils, in combination with soil physical properties (Chapin & Bliss, 1986; Michalet et al., 2002), is recognized as a primary driver of vegetation assemblage composition (Gough et al., 2000; Jarvis & Hatch, 1986; Schaffers & Sýkora, 2000; van der Welle et al., 2003). Consequently, calcareous soils generally harbor floristically distinct vegetation communities compared with acidic soils, even where such soil systems are adjacent (Ström, 1997; Tansley, 1917).

Most studies have focused on how calcifuge plant strategies influence the natural distribution of species or vegetation communities across different soil systems, and the physiological determinants of occurrence on acidic or calcareous soils (e.g., De Silva, 1934; Etherington, 1981; Fühner & Runge, 2009; Grubb et al., 1969; Hayes et al., 2019; Tansley, 1917; Tyler & Olsson, 1993). One context in which calcifuge plant strategies have not been adequately considered, yet which may be of huge economic and environmental significance, is in the establishment of vegetation on degraded, heavily altered, or reconstructed substrates through rehabilitation or ecological restoration. The reinstatement of representative native vegetation on degraded, heavily altered, or reconstructed substrates may be challenging, if edaphic conditions contrast starkly with those of undisturbed soils harboring native reference communities (Cross et al., 2018, 2019). Native plant communities are generally adapted to local hydrological and geochemical conditions, particularly in geologically ancient and climatically buffered landscapes such as southwestern Australia (Hopper, 2009; Hopper et al., 2016) and southeastern Brazil (Do Carmo & Jacobi, 2016; Furley, 1999; Silveira et al., 2016). Many vegetation communities in these regions are adapted to heavily weathered, acidic, and nutrient‐impoverished soils (Cross & Lambers, 2017) and have developed in close association with shifts in nutrient dynamics and innate edaphic constraints along soil chronosequences over hundreds, thousands, or even millions of years (Laliberté et al., 2012; Turner & Laliberté, 2015; Walker & Syers, 1976; Wardle et al., 2004).

The return of functional, biodiverse native plant communities following ecosystem degradation by activities such as mining is often desired or required in regions supporting significant extractive industries such as Western Australia and Brazil (Stevens & Dixon, 2017; Cross et al., 2017). The substrates produced by mining activities often exhibit geochemical conditions that differ from those of adjacent undisturbed soils (i.e., contamination with heavy metals and other deleterious compounds, a lack of organic matter and nutrients, and altered hydrological or physical properties) and are frequently characterized by heavily altered pH conditions (e.g., Cross et al., 2017; Hudson‐Edwards et al., 2011; Jamieson, 2011; Wong, 2003; Yong‐Zhong et al., 2005). Chemical discharge, the processing of mineral ores, and fertilizer inputs can result in highly acidic substrates with pH values as low as 2–3 (e.g., Dowarah et al., 2009; Glamore & Indraratna, 2004; Johnston et al., 2014; Juwarkar & Jambhulkar, 2008; Rieder et al., 2013), while highly processed mined materials such as magnetite tailings can be extremely alkaline (pH 10 or higher; Mishra et al., 2004; Oliveira et al., 2005; Wehr et al., 2006; Adcock et al., 2007; Cross & Lambers, 2017). Calcicole and calcifuge groups, as well as species with specialized nutrient‐acquisition strategies more typical of ancient, heavily weathered soils than young, unweathered soils (e.g., species with arbuscular, ericoid, or ectomycorrhizal associations and species producing cluster roots; Lambers & Oliveira, 2019), may be strongly selected against on heavily altered substrates which may compromise efforts to rehabilitate or restore degraded landscapes (Cross & Lambers, 2017; Cross et al., 2018, 2019).

Calcicole and calcifuge plant strategies, as well as nutrient‐acquisition strategies, will likely act as a filter to species establishment in ecological restoration on heavily altered or reconstructed substrates. However, we cannot currently predict the degree to which this filter might affect restoration outcomes, as there is little evidence for how patterns of these strategies are expressed by vegetation communities and regional floras across landscapes. This study provides the first such examination and presents an analysis of calcifuge plant strategies (using the calcifuge index developed by Grime and Lloyd (1973)) and nutrient‐acquisition strategies in 538 plant species from 830 relevés in 29 previously classified vegetation communities on different soil types in an area of >82,000 ha in the semiarid Mid‐West region of Western Australia. Vegetation communities in the study site range from open halophytic scrub on saline flats to dense tall shrubland on the skeletal soils of ancient Banded Ironstone Formations (BIF) ridgelines, and BIF in particular are recognized for harboring high levels of floristic biodiversity (Payne et al., 1998; Markey and Dillon, 2008). Vegetation communities on BIF are likely edaphically determined, with edaphic isolation and the physical constraints of extremely shallow soils selecting for “edaphic endemics” at increasing elevation on these landforms (Gibson et al., 2010; Poot & Lambers, 2003). We examined to what extent soil chemistry and landscape position influenced plant distribution and calcifuge plant strategies in the region, aiming specifically to determine: i) whether relevé plant species richness was predicted by landscape position, elevation, or soil chemistry factors including pH, salinity (electrical conductivity [EC] and sodium [Na]), macronutrients (total N, P, Ca, and K), and micronutrients important for plant growth and development (Fe and Ca); ii) whether relevés from classified vegetation communities exhibited different soil chemistry conditions to support edaphic factors as a driver of floristic turnover in the region; iii) the proportion of calcifuge, soil‐indifferent, and calcicole species in the regional flora, as well as the proportion of species with different nutrient‐acquisition strategies, and whether these proportions varied among different vegetation communities and landscape position; and iv) whether calcifuge plant strategy or nutrient‐acquisition strategy was predicted by landscape position, elevation, or soil chemistry factors, to determine the degree to which these strategies might act as filters for ecological restoration in the region.

## 2 METHODS

### 2.1 Study site

The study site is an 82,000‐ha area (up to 22 km east‐west and 14 km north‐south) of predominantly intact remnant native vegetation centered on a major magnetite mining operation, located approximately 400 km northeast of Perth, in the Mid‐West region of Western Australia (29°13′1″S, 116°41′13″E). The area lies on the boundary between the extra‐dry and semidesert Mediterranean regions and experiences a semiarid dry Mediterranean climate with mild winters (mean monthly maximum 19°C) and hot, dry summers (mean monthly maximum 37°C. Since 1991, the site has received an average of 303 ± 13.3 mm annual rainfall (minimum 168.8 mm, maximum 466.6 mm), predominantly falling in winter between May and September (Australian Bureau of Meteorology, Station 10,195, www.bom.gov.au).

### 2.2 Soils and soil analyses

**FIGURE 1:**
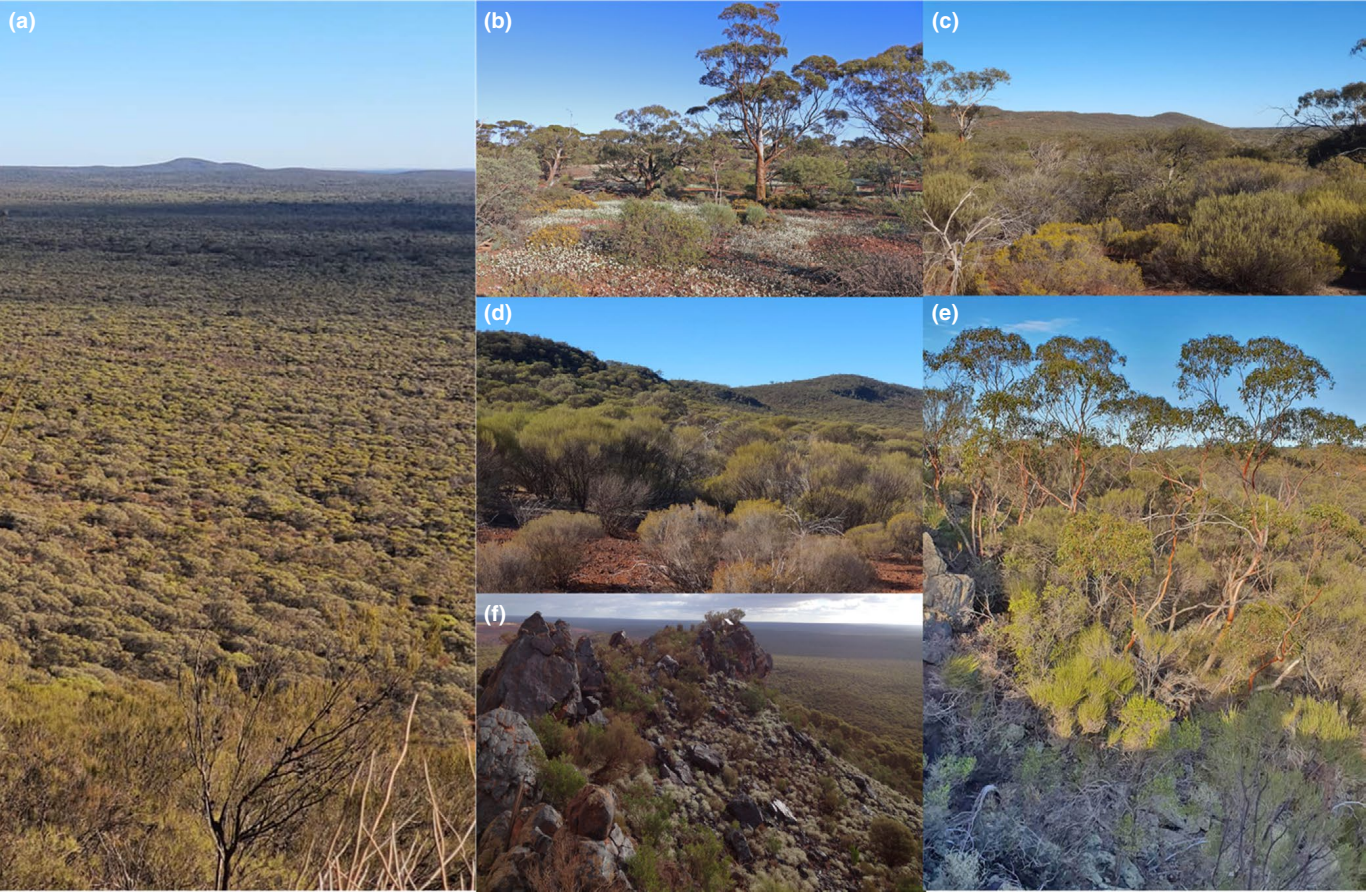
The study site predominantly comprises gently undulating plains broken by ridges of metamorphic rock (a). Vegetation on the undulating plains predominantly varies from open *Eucalyptus* woodland over mixed shrubs and annual herbs on deep red earths (b) to *Acacia*‐dominated shrubland on shallow stony soils (c); the slopes of ridges typically harbor dense transitional shrubland dominated by species of *Acacia* and *Allocasuarina* on shallow stony soils (d); and ridges typically harbor dense tall shrubland dominated by species of *Acacia*, *Eremophila,* and *Philotheca* and occasionally emergent *Eucalyptus* (e), with dense low mixed shrubland among exposed rocky outcrops occurring along crests (f). Photographs by A. Freeman (a, c–e), S. Cross (b), and A. Cross (f)

The landscape of the study site comprises predominantly ancient granitic undulating plains broken by ridges of metamorphic rocks such as banded ironstone (Figure 1) (Payne et al., 1998). Twelve broad soil groups have been classified from within the study area, the most extensive being sands on sandplains and granitic country, and variable depth red earths overlying a hardpan on level to gently inclined plains (Payne et al., 1998). Soils are generally massive, occasionally texture‐contrast, and range from calcareous and saline on lower flood plains, to skeletal, acidic, and highly weathered on ridgelines and upper slopes of BIF ranges (Payne et al., 1998; Landloch, 2006). A total of 24 discrete land systems have been identified from the study site (Payne et al., 1998), with land systems defined as areas with recurring patterns of topography, soils, and vegetation (Christian & Stewart, 1953).

In order to provide a representative sample of soil chemistry across the study site, five replicate 400 g soil samples were collected from the upper 50 mm in open, unvegetated, and undisturbed areas from each of the 24 identified land systems (120 samples total). Replicates from each land system were collected no closer than 100 m from one another and from at least two discrete areas of each particular land system, in an attempt to capture as much natural variability as possible. All substrate samples were stored dry in canvas bags for a week prior to analysis of pH (H_2_O), EC, total N, Colwell P and K, Fe, Ca, and Na (ChemCentre, Perth, Australia). Data were supplemented with previously published soil chemistry from 41 soil sampling locations across the 24 land systems (Landloch, 2006). Mean values for species richness (per 400 m^2^ relevé) pH, EC (S/m), [N] (mg/kg), [P] (mg/kg), [K] (mg/kg), [Fe] (mg/kg), [Ca] (mg/kg and mmol/dm^3^), and [Na] (mg/kg) were determined for all land systems, with samples >3 standard deviations from the mean removed from analyses (only one sample was removed). While we acknowledge that analyzed soil chemistry data are a generalized examination of regional soils, they are intended to be representative, and it was logistically unfeasible to undertake more comprehensive sampling given the large area (>82,000 ha), poor accessibility, and remoteness of the study site.

### 2.3 Vegetation data

Given its location on the extra‐dry and semidesert Mediterranean region boundary, the study site exhibits a transitional flora characterized by components of both the South‐West Botanical Province and the Eremaean Botanical Province (Beard, 1990). Local vegetation communities are relatively typical of Eremaean sclerophyll shrubland (Figure 1) (Beard, 1990) and most commonly include low to open woodlands, predominantly comprising shrubs or trees from genera including *Acacia*. (e.g., *A. sibina, A. ramulosa* var. *ramulosa*), *Eucalyptus* (e.g., *E. leptopoda*, *E. kochii*), and *Melaleuca* (e.g., *M. hamata* and *M*. *leiocarpa*), often with *Allocasuarina acutivalvis*, *Callitris columellaris*, *Hakea recurva* subsp. *recurva*, and an understorey of shrubs, grasses and herbaceous annuals (Markey and Dillon, 2008). The floristic biodiversity of the region is well documented, with broad vegetation descriptions provided by Beard (1990) and Payne et al. (1998), and detailed vegetation surveys of the study site undertaken in 2003 (Bennett Environmental Consulting, 2004), 2007 (Markey and Dillon, 2008), and 2008–2011 (Woodman Environmental Consulting, 2012), providing 830 relevés across all 24 land systems that were analyzed in this study. All relevés, each 20 m × 20 m, were grouped into one of three landscape positions (plains, slopes, and crests and ridges) as previous phytosociological analyses indicated that the vegetation of the study site is most parsimoniously classified by landscape position (Woodman Environmental 2004). Elevation was recorded for each relevé in addition to species presence/absence and species richness, and all relevés were allocated the mean values of pH, EC, [N], [P], [K], [Fe], [Ca], and [Na] for their particular land system. All 538 plant species were then assigned mean, maximum and minimum values for pH, EC, [N], [P], [K], [Fe], [Ca], [Na], and elevation, calculated from all relevés in which each species occurred, as well as classes for life history (the longevity of individuals of each species season to season; annual, perennial, geophyte) and nutrient‐acquisition guild (Appendix S1). Nutrient‐acquisition guilds were assigned following Lambers and Oliveira (2019), Brundrett (2009) and Cross et al. (2019), and included nonmycorrhizal (NM), arbuscular mycorrhizal (AM), AM/ectomycorrhizal (AM/ECM), AM/N_2_‐fixing and AM/ECM/N_2_‐fixing (NF), nonmycorrhizal with cluster roots (CR), varied (AM/ECM/NF/CR), and species with other highly specialized strategies such as carnivorous plants, orchids, and holo‐ and hemiparasites (other).

To facilitate analysis of calcifuge plant strategies, all 538 species were assigned a calcifuge index (*IC*), calculated from histograms of species constancy in 0.5 pH unit classes sensu Grime and Lloyd (1973) and Etherington (1981). Values of the index were calculated as $IC=\;\frac{\mathrm{constancy}\;\mathrm{in}\;\mathrm{classes}\;\mathrm{of}\;\mathrm{pH}<5.5\;}{\mathrm{constancy}\;\mathrm{in}\;\mathrm{all}\;\mathrm{pH}\;\mathrm{classes}}\times 100$. Species with *IC* values of 100 were classified as “*likely calcifuge*,” 75–99 as “*possibly calcifuge*,” 26–74 as “*soil indifferent*,” 1–25 as “*likely not calcifuge*,” and 0 as “*highly* *likely not calcifuge*.” Species were classified as “not calcifuge” rather than “calcicole” from *IC,* as soil exchangeable Ca, rather than soil pH, is the determining factor for calcicole species (De Silva, 1934; Gao et al., 2020). Probability of occurrence curves was generated for each classified calcifuge strategy class for selected soil parameters (mean soil pH, [Ca], elevation, total [N], total [P], and [K]), reflecting the probability of a species from that class occurring at any given value of each soil parameter.

### 2.4 Univariate statistical analyses

One‐way MANOVA with Tukey post hoc tests (with Bonferroni correction; SPSS Statistics 26, IBM, New York, USA) was used to examine whether relevé fixed factors (soil pH, EC, N, P, K, Fe, Ca, Na, elevation, and landscape position) varied significantly among the three landscape positions (plains, slopes, and crests and ridges) and among the five classified calcifuge strategies (*likely calcifuge*, *possibly calcifuge*, *soil indifferent*, *likely not calcifuge,* or *highly likely not calcifuge*). One‐way MANOVA with Tukey post hoc tests was also used to examine whether proportion of calcifuge strategies (percentage of total species recorded from relevés that were *likely calcifuge*, *possibly calcifuge*, *soil indifferent*, *likely not calcifuge,* or *highly likely not calcifuge*), and the proportion of plant nutrient‐acquisition strategies (percentage of total species recorded from relevé that were NM, AM, AM/ECM, NF, CR, AM/ECM/NF/CR, or other), varied significantly among the three landscape positions (plains, slopes, and crests and ridges). Pillai's Trace (robust and recommended when sample sizes are unequal yielding a statistically significant Box's M result) was utilized as the test statistic as covariance matrices were unequal for analyses of relevé fixed factors (Box's *M* = 2,908.2, *F* = 3.562, *p* < .001) and species proportions by landscape position (Box's *M* = 278.9, *F* = 18.467, *p* < .001). Bivariate Pearson correlations were employed to examine whether relevé species richness was correlated with the proportion of calcifuge strategies in each relevé.

Generalized linear models with a Poisson probability distribution and a log link function (GLM, SPSS Statistics 26, IBM, New York, USA) were used to test the main effects of relevé fixed factors (landscape position) and covariates (soil pH, EC, total [N], total [P], [K], [Fe], [Ca], [Na], elevation) on relevé species richness.

Generalized linear mixed‐effect models (GLMM, SPSS Statistics 26, IBM, New York, USA) were used to test the main effects of species fixed factors (mean, maximum, and minimum values of soil pH, EC, total [N], total [P], [K], [Fe], [Ca], [Na], and elevation, as well as life history and nutrient‐acquisition strategy) on *IC*, with model parameters subject to species‐ and family‐specific random effects and conditional pairwise comparison tests run to provide contrasts within factors, adjusted for random effects. The inclusion of random effect terms was tested through a likelihood‐ratio test, derived from the maximum‐likelihood measure of competing models. A multinomial logistic regression model (SPSS Statistics 26, IBM, New York, USA), with AM as the reference category, was fitted to test the main effects of species fixed factors (mean, maximum, and minimum values of soil pH, EC, total [N], total [P], [K], [Fe], [Ca], [Na], and elevation) on the seven nutrient‐acquisition classes. All fixed factor predictors with variance inflation factor >10 were removed from GLMM and multinomial logistic regression models, and stepwise model reduction was completed through minimizing the Akaike's information criterion (AIC).

### 2.5 Multivariate statistical analyses

All multivariate analyses were undertaken in Primer 7 (Primer‐e, Auckland, NZ), using a relevé × environmental variable matrix (830 relevés × 11 environmental variables), a relevé × species matrix (830 relevés × 538 species), a relevé × proportion of classified calcifuge strategies (percentage of total species recorded from relevé that were *likely calcifuge*, *possibly calcifuge*, *soil indifferent*, *likely not calcifuge,* or *highly likely not calcifuge*) matrix (830 relevés × 5 calcifuge classes), and a relevé × proportion of nutrient‐acquisition strategies (percentage of total species recorded from relevé that were NM, AM, AM/ECM, NF, CR, AM/ECM/NF/CR, or other) matrix (830 relevés × 538 species). Species accumulation curves were generated using Chao 2 with 999 permutations.

BEST analysis was used to determine which environmental variables (soil pH, EC, total [N], total [P], [K], [Fe], [Ca], [Na], and elevation) most parsimoniously explained observed patterns of relevé species composition, the proportions of classified calcifuge strategies, and the proportions of nutrient‐acquisition strategies, through maximization of a rank correlation between relevé × environmental variable (Euclidean distance; Clarke and Gorley, 2006) with either relevé × species (Bray–Curtis similarity; Clarke and Gorley, 2006), relevé × proportion of classified calcifuge strategies (Bray–Curtis similarity), or relevé × proportion of nutrient‐acquisition strategies (Bray–Curtis similarity) resemblance matrices. Nonmetric multidimensional scaling (nMDS) was used to visualize the ordination of relevés between resemblance matrices of environmental variables and species composition, environmental variables and proportions of classified calcifuge strategies, and environmental variables and proportions of nutrient‐acquisition strategies.

The level of dissimilarity in environmental variables, plant species composition, proportions of classified calcifuge strategies, and proportions of nutrient‐acquisition strategies between landscape position groups was assessed using analysis of similarity (ANOSIM), which generates an R‐statistic value from −1 to 1 with higher values indicating a greater compositional dissimilarity among groups (Clarke and Warwick, 2001). The percentage contribution of indicator parameters, species, and classes to dissimilarity between groups was determined using similarity percentages (SIMPER), based on Euclidean (environmental parameters) and Bray–Curtis resemblance (plant species composition, proportions of classified calcifuge strategies, and proportions of nutrient‐acquisition strategies) matrices.

Distance‐based linear models (DistLM, using the PERMANOVA + extension to Primer 7) with stepwise model selection using AIC were used to derive the most parsimonious models predicting patterns of relevé species composition, patterns of classified calcifuge strategies (excluding soil pH as a variable), and patterns of nutrient‐acquisition strategies, using normalized environmental variables. DistLM identifies significant contributing predictor variables and the variation explained by each predictor, and distance‐based redundancy analysis (dbRDA) models were produced to visualize the relative contributions of predictor variables to observed patterns in each analysis.

All statistical tests were conducted using 95% CI, with significance determined by *p* < .05. Data are presented as means ± standard error of the raw data, unless stated otherwise.

## 3 RESULTS

### 3.1 Patterns and predictors of soil chemistry

**FIGURE 2:**
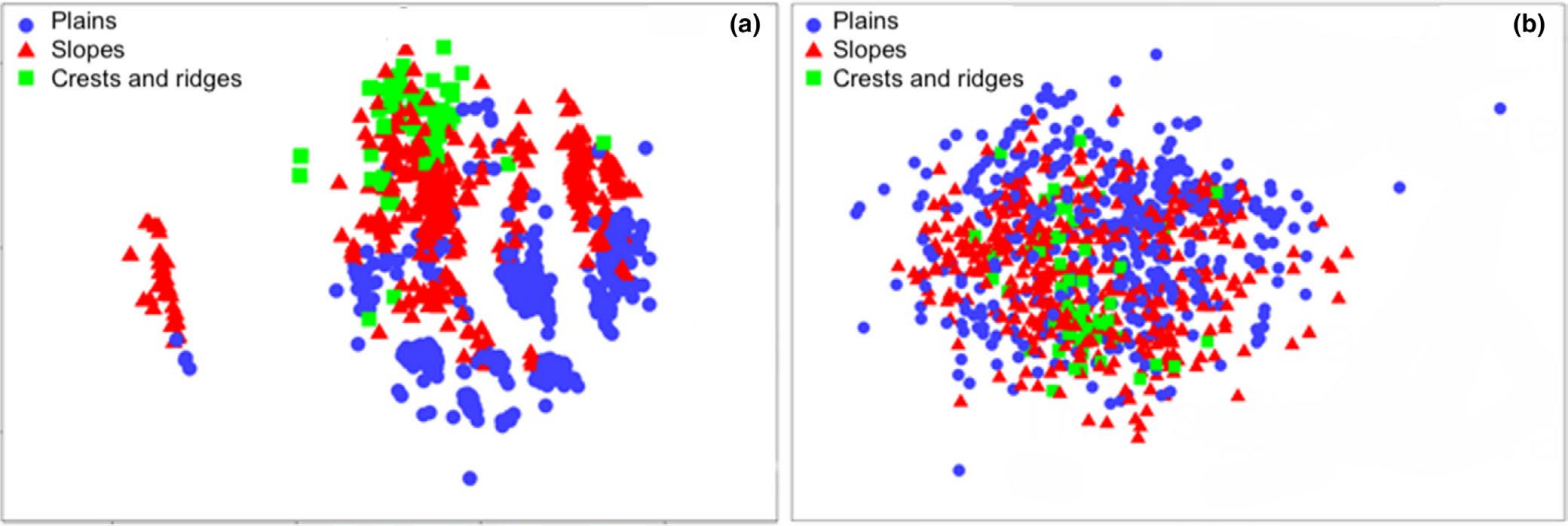
nMDS ordination of 830 relevés from different landscape positions in the Mid‐West region of Western Australia, on the basis of environmental variables (a) and plant species composition (b)

One‐way MANOVA indicated that there was a significant effect of landscape position on the combined relevé variables, *F*(18, 1,640) = 27.330, *p* < .001, Pillai's Trace = 0.461, partial *η* ^2^ = 0.231, and relevés clustered markedly along a broad landscape position gradient (Figure 2a). The soil of relevés on plains and slopes was generally slightly acidic (occasionally neutral to alkaline) and often slightly saline, while soil from crests and ridges was acidic and exhibited low EC (Table 1). Soil [Fe], [Na], and total [P] decreased along an increasing elevation gradient, [Ca] increased along the same gradient, and total [N] and [K] were highest in the soil of slopes relevés (Table 1). Consequently, ratios of N:P:K varied markedly from approximately 1:5:5 in the soil of plains relevés to 1:1.5:3 in the soil of slopes and 1:2:4 in the soil of crests and ridges. Soil exchangeable [Ca] across the study site ranged from 0.5 to 51.2 mM (mean 10.7 mmol/dm^3^).

**TABLE 1:** Mean ± *SE*, with ranges in parentheses, for floristic species richness and soil parameters of relevés on plains (*n* = 383), slopes (*n* = 385), and crests and ridges (*n* = 62) in the Mid‐West region of Western Australia

Parameter	Plains	Slopes	Crests and ridges
Species richness	17 ± 0.5 (2–70)^a^	22 ± 0.7 (4–72)^b^	35 ± 2.2 (9–64)^c^
pH	6.4 ± 0.06 (4.4–8.5)^a^	5.8 ± 0.06 (4.7–8.1)^b^	4.8 ± 0.05 (4.7–6.5)^c^
EC (S/m)	6.2 ± 0.27 (0.1–16.6)^a^	5.8 ± 0.31 (0.2–16.6)^a^	1.5 ± 0.37 (0.2–12.5)^b^
[Na] (mg/kg)	64 ± 2.6 (1.2–170.0)^a^	57 ± 1.9 (3.2–160.0)^b^	29 ± 1.7 (3.2–116.7)^c^
Total [N] (mg/kg)	31 ± 4.1 (3.1–470.0)^a^	72 ± 7.4 (3.5–470.0)^b^	34 ± 7.8 (3.8–220.0)^a^
Total [P] (mg/kg)	158 ± 4.2 (4.7–405.0)^a^	103 ± 4.1 (4.7–310)^b^	63 ± 6.6 (5.0–310.0)^c^
[K] (mg/kg)	146 ± 9.2 (13.3–1,433.3)^a^	238 ± 20.5 (13.3–1,433.3)^b^	139 ± 6.8 (26.7–340)^a^
[Ca] (mg/kg)	151 ± 7.6 (10.0–1,027.6)^a^	269 ± 13.7 (10.0–1,027.6)^b^	246 ± 14.8 (26.7–787.0)^b^
[Ca] (mmol/dm^3^)	8 ± 0.4 (0.3–25.6)^a^	13 ± 0.7 (0.3–25.6)^b^	12 ± 0.7 (0.6–19.6)^b^
[Fe] (mg/kg)	96 ± 3.6 (10.8–188.7)^a^	83 ± 2.7 (15.5–188.7)^b^	59 ± 3.0 (15.3–188.7)^c^
Elevation (m a.s.l.)	358 ± 1.2 (291–424)^a^	378 ± 1.4 (302–461)^b^	384 ± 3.8 (320–454)^c^

Note

All relevés were 400 m^2^. Annotated lettering represents the results of conditional pairwise comparison tests (one‐way MANOVA with Tukey HSD, contrasts within each species). Values followed by the same or no letters are not significantly different among sites for each characteristic (at *p* <.05). a.s.l. = above sea level.

ANOSIM indicated a high level of dissimilarity in the environmental variables among landscape positions (*R* = 0.261, *p* = .001), driven primarily by strong dissimilarity among plains and crest and ridges (R = 0.763, *p* = .001) and among plains and slopes (*R* = 0.219, *p* = .001). The soils of slopes and crests and ridges were marginally dissimilar (*R* = 0.056, *p* = .05). Variables contributing most significantly to landscape position dissimilarities were [Ca], total [P], [K], total [N], [Fe], and soil pH among plains and slopes, total [P], soil pH, [Na], and [Fe] among plains and crest and ridges, and EC, total [N], [K], and [Ca] among slopes and crest and ridges (factor contributions between 9% and 12% in all three cases).

### 3.2 Patterns and predictors of floristic composition and species richness

**FIGURE 3:**
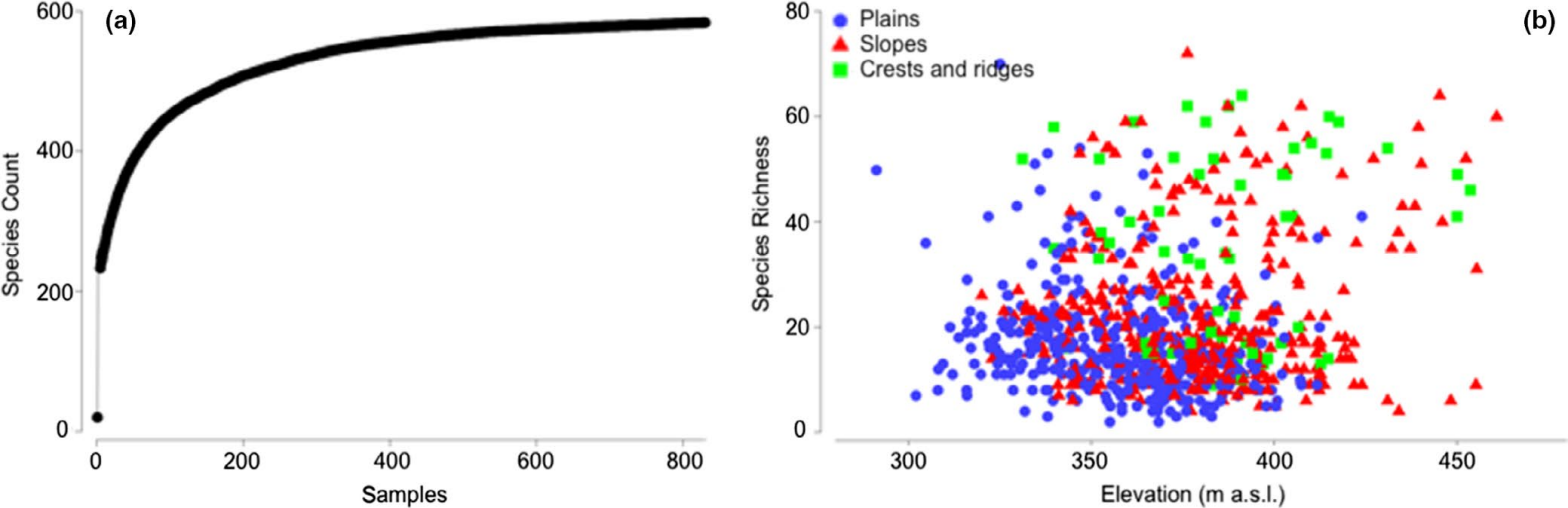
Species accumulation curve (a) and scatterplot of relevé species richness by elevation (b) clustered by landscape position (blue—plains, red—slopes, and green—crests and ridges). m a.s.l.: meters above sea level

The 830 analyzed relevés comprised 17,158 records of 538 native plant species from 204 genera in 64 families, with number of records per species ranging from 1 to 438 (mean 32 ± 2.3 records). Species accumulation curves indicated that 50% of regional species richness was obtained from 12 random relevés (Figure 3a), while 90% and 99% of regional species richness were obtained from 149 and 282 random relevés, respectively. The most commonly represented families included Asteraceae (82 species), Fabaceae (61), Myrtaceae (45), Chenopodiaceae (37), Poaceae (27), and Proteaceae (21). The richest genera included *Acacia* (Fabaceae, 40 species), *Eremophila* (Scrophulariaceae, 18), *Calandrinia* (Portulacaceae, 17), *Rhodanthe* (Asteraceae, 15), *Eucalyptus* (Myrtaceae, 13), *Grevillea* (Proteaceae, 12), *Maireana* (Chenopodiaceae, 12), *Melaleuca* (Myrtaceae, 12), and *Ptilotus* (Amaranthaceae, 12).

Species richness per 400 m^2^ relevé ranged from 2 to 72 (mean 21 ± 0.5 species) and was greatest in crest and ridgeline habitats higher in the landscape (Figure 3b) where soil total [P], [Na], and [Fe] were lowest (Table 1). Relevé species richness was significantly predicted by a GLM including soil pH, total [N], total [P], [K], [Fe], [Ca], [Na], elevation, and landscape position. Species richness was negatively correlated with soil total [P], [Fe], soil pH, soil total [N], and [Na] and positively correlated with soil [Ca], elevation, and soil [K] (Table 2).

**TABLE 2:** Parameter estimates from GLM fitted to test the main effects of relevé fixed factors (landscape position) and covariates (soil pH, EC, total [N], total [P], [K], [Fe], [Ca], [Na], elevation) on floristic species richness from 830 relevés in the Mid‐West region of Western Australia

Parameter	Estimate (B)	Standard error	*df*	Wald chi‐square	*p*	95% confidence interval	
						Lower bound	Upper bound
*Mean total [P]*	−0.002	0.0002	1	128.81	<.001	−0.002	−0.001
*Landscape position*	−0.335	0.0309	1	118.04	<.001	−0.396	−0.275
*Mean [Fe]*	−0.002	0.0002	1	96.733	<.001	−0.003	−0.002
*Mean pH*	−0.125	0.0129	1	93.268	<.001	−0.150	−0.099
*Mean total [N]*	−0.002	0.0003	1	73.125	<.001	−0.003	−0.002
*Mean [Na]*	−0.002	0.0003	1	37.42	<.001	−0.002	0.001
*Mean [Ca]*	0.001	0.0001	1	23.261	<.001	0.000	0.001
*Elevation*	0.001	0.0003	1	14.327	<.001	−0.002	0.001
*Mean [K]*	0.001	0.0001	1	6.029	.014	−0.001	0.001

**FIGURE 4:**
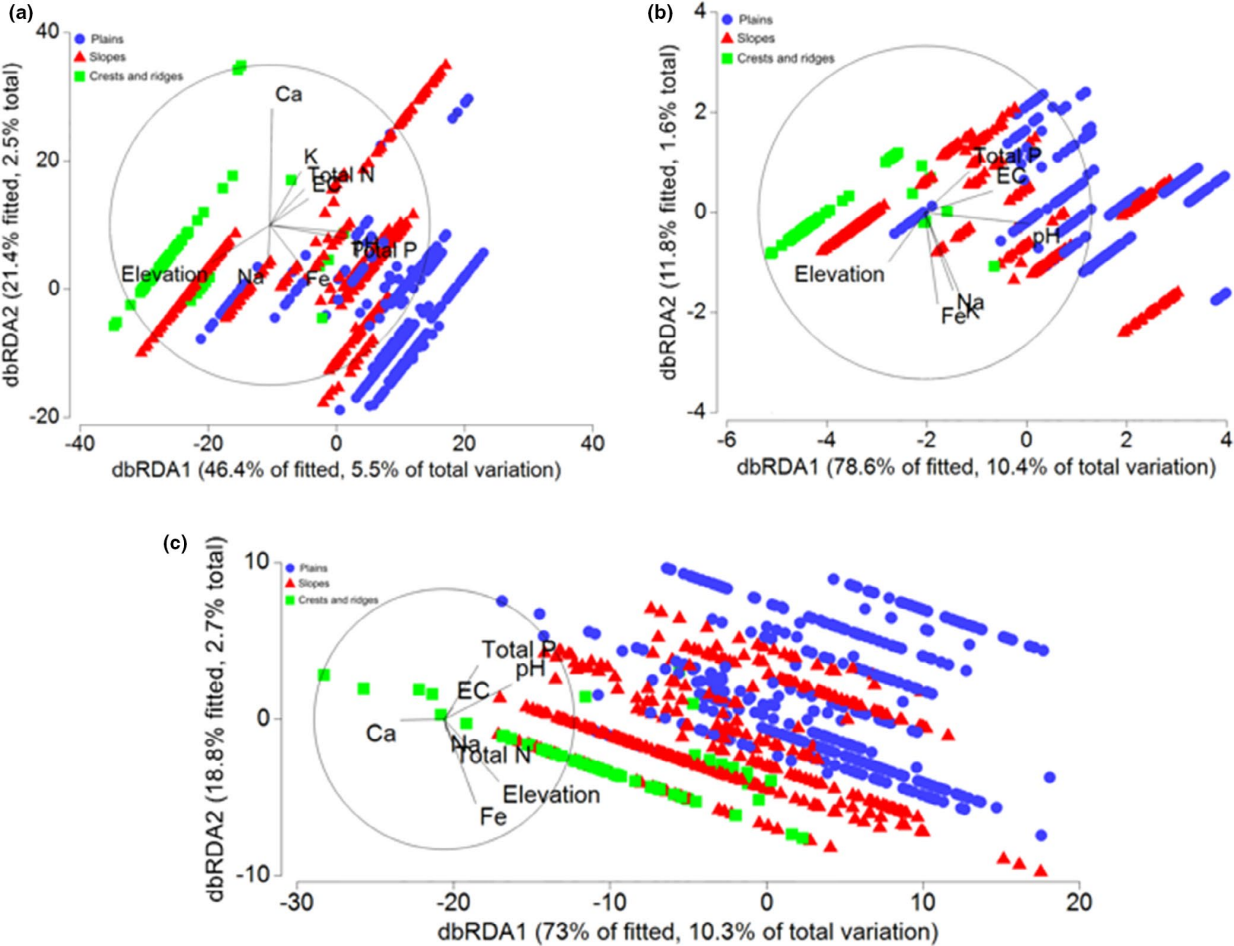
Distance‐based redundancy analysis (dbRDA) models visualizing the relative contributions of predictor variables to observed patterns in species composition (a), the proportion of calcifuge plant strategies (b), and the proportion of nutrient‐acquisition strategies (c) in 830 relevés from the Mid‐West region of Western Australia

BEST analyses indicated the most parsimonious environmental variable selection explaining the observed patterns of relevé plant species composition included landscape position, soil pH, [Ca], [Fe], and elevation (Pearson *R* = 0.170). No informative patterns were evident from nMDS ordinating relevés by species composition (Figure 2b), but ANOSIM indicated there was a significant difference in species composition among landscape positions (*R* = 0.062, *p* = .001). The species composition of plains relevés differed significantly from the composition of relevés on slopes (*R* = 0.077, *p* = .001) and on crests and ridges (*R* = 0.087, *p* = .004), while the vegetation of slopes and crests and ridges was compositionally very similar (*R* = −0.083, *p* = .999). The most parsimonious DistLM explained 11.7% of variation in species composition (Table 3) and included landscape position, [Ca], elevation, soil pH, [Na], [Fe], total [N], EC, total [P], and [K] as significant predictors (Figure 4a).

**TABLE 3:** Variables and statistics from most parsimonious DistLM models explaining variation in species composition and the proportion of calcifuge plant strategies and nutrient‐acquisition strategies for 538 species from the Mid‐West region of Western Australia

Factor	Variable	% variation explained	Pseudo‐F	*p*
Species composition	Landscape position	2.9	25.1	<.001
	[Ca]	1.8	15.2	<.001
	Elevation	1.6	14.6	<.001
	Soil pH	1.3	11.9	<.001
	[Na]	0.9	8.6	<.001
	[Fe]	0.9	7.7	<.001
	Total [N]	0.8	7.5	<.001
	EC	0.6	6.0	<.001
	Total [P]	0.6	5.5	<.001
	[K]	0.2	2.2	.002
Calcifuge plant strategies	Landscape position	4.1	35.1	<.001
	EC	2.8	25.1	<.001
	[K]	1.9	18.1	<.001
	[Fe]	1.0	9.4	<.001
	Total [P]	0.8	6.9	<.001
	[Na]	0.7	7.1	.002
	Elevation	0.4	3.3	.035
	Total [N]	0.3	3.1	.039
Nutrient‐acquisition strategy	Landscape position	3.6	30.9	<.001
	Elevation	2.7	23.8	<.001
	Soil pH	2.3	21.5	<.001
	[Ca]	2.1	18.7	<.001
	[Fe]	1.4	12.9	<.001
	[K]	0.6	5.8	.001
	Total [P]	0.5	5.1	.013
	EC	0.4	3.9	.013
	Total [N]	0.4	3.9	

### 3.3 Patterns and predictors of calcifuge plant strategies

**FIGURE 5:**
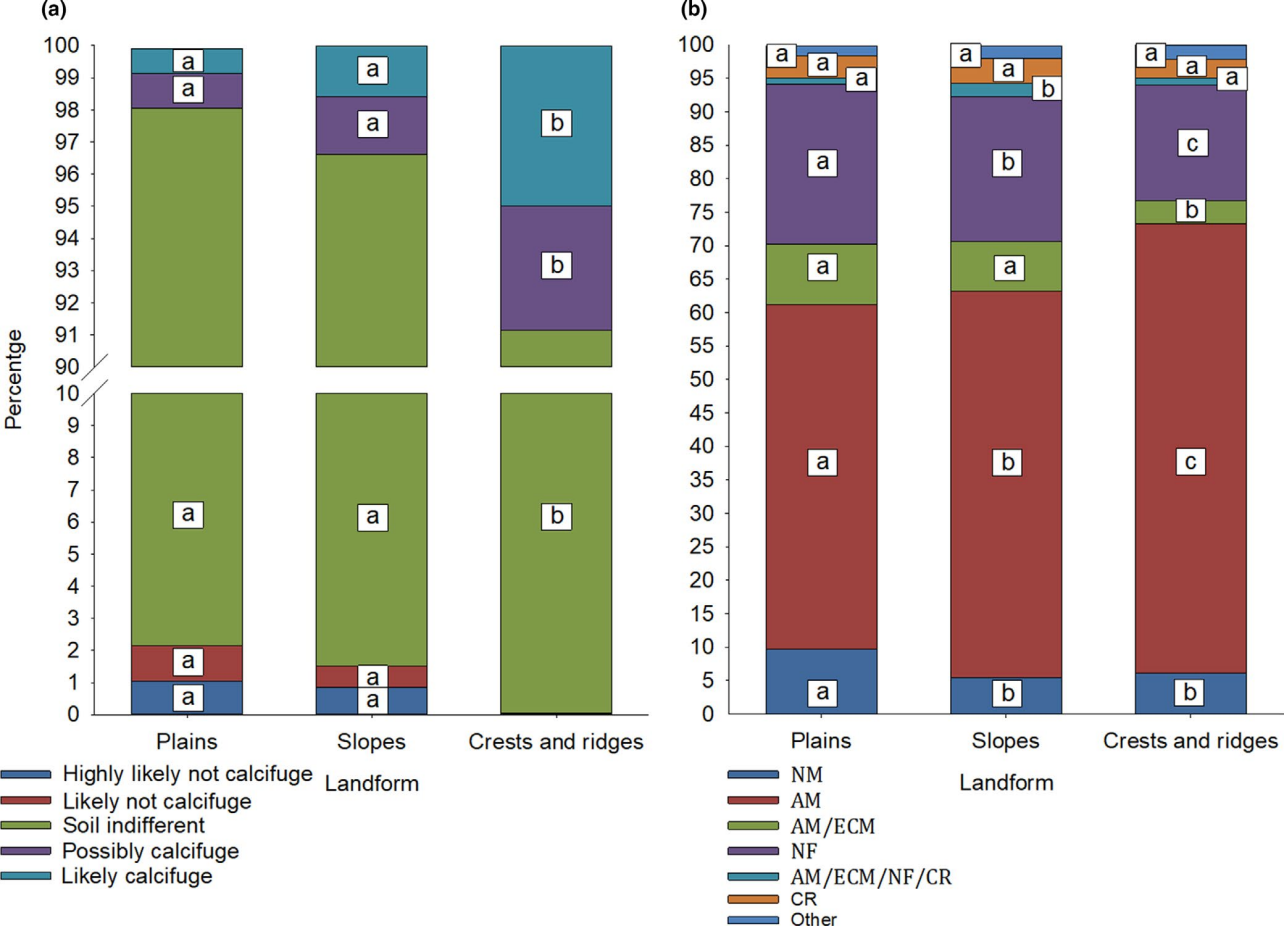
Proportion of species from each of the classified calcifuge strategies (a) and nutrient‐acquisition strategies (b) present in vegetation communities of three landscape positions from 830 relevés in the Mid‐West region of Western Australia, as a percentage of total species richness. Annotated lettering represents the results of conditional pairwise comparison tests (one‐way MANOVA with Tukey HSD, contrasts among landscape position). Values followed by the same or no letters are not significantly different among landscape position for each characteristic (at *p* < .05)

*IC* values for the 538 species ranged from 100 to 0, with a mean of 50.5 ± 1.28. A total of 106 species were classified as *likely calcifuge* (19.9%), 34 species as *possibly calcifuge* (6.3%), 332 species as *soil indifferent* (61.6%), 13 species as *likely not calcifuge* (2.4%), and 53 species as *highly likely not calcifuge* (9.8%). One‐way MANOVA indicated there was no significant difference between classified vegetation communities on the proportion of calcifuge plant strategies, *F*(145, 4,000) = 1.036, *p* = .368, Pillai's Trace = 0.181, partial η^2^ = 0.036, but that the proportion of calcifuge plant strategies did vary significantly among landscape positions, *F*(10, 1648) = 10.748, *p* < .001, Pillai's Trace = 0.122, partial η^2^ = 0.061 (Figure 1). *Soil‐indifferent* species comprised the vast majority of species records from relevés in all landscape position categories (95.2 ± 0.23% across the entire study site), but the proportion of these species was significantly lower (91.1 ± 0.83%) in crest and ridgeline relevés. The proportion of *highly likely not calcifuge* and *likely not calcifuge* species decreased along a landscape position gradient from 1.2% and 1.1%, respectively, in plains relevés to virtually nil in crest and ridgeline relevés. This contrasted with an increase in the proportion of *likely calcifuge* and *possibly calcifuge* species along the same gradient, from 0.8% and 1.1%, respectively, in plains relevés to 5.0% and 3.8%, respectively, in crest and ridgeline relevés (Figure 5a).

The most parsimonious GLMM predicting *IC* included soil [Ca], [K], [Na], total [N], and total [P], minimum pH and [Na], and maximum pH as highly significant parameters (Table 4), with *IC* negatively correlated with all significant parameters. Almost all records (>99%) of species with *IC* >75 were recorded from soil with <7.5 mmol/dm^3^ exchangeable [Ca], and none occurred in soil exceeding 10 mmol/dm^3^ exchangeable [Ca]. Species with *IC* <25 typically occurred in soil with exchangeable [Ca] between 7.5 and 25 mmol/dm^3^.

**TABLE 4:** Parameter estimates from the most parsimonious GLMM fitted to test the main effects of species fixed factors (mean, maximum, and minimum values of pH, EC, total [N], total [P], [K], [Fe], [Ca] (mg/kg), Ca (mmol/dm^3^), [Na], and elevation, as well as life history, nutrient‐acquisition strategy, and plant family) on index of calcifugy (*IC*) for 538 species from the Mid‐West region of Western Australia

Parameter	Estimate	Standard error	*df*	*t*	*p*	95% confidence interval	
						Lower bound	Upper bound
*Minimum pH*	−15.1202	1.1460	437.509	−13.190	<.001	−17.3725	−12.8678
*Maximum pH*	−19.1510	0.5858	528.320	−32.680	<.001	−20.3019	−18.0000
*Mean [Ca]*	0.0503	0.0091	481.876	5.477	<.001	0.0322	0.0683
*Mean [K]*	−0.0552	0.0104	425.726	−5.272	<.001	−0.0758	−0.0346
*Mean [Na]*	−0.1618	0.0327	496.600	−4.943	<.001	−0.2262	−0.0975
*Minimum [Na]*	−0.01467	0.0316	458.085	−4.639	<.001	−0.2088	−0.0845
*Mean total [N]*	0.0591	0.0195	507.562	3.018	.003	0.0206	0.0975
*Mean total [P]*	0.0627	0.0123	516.954	5.076	<.001	0.0384	0.0870
*(Species)*	92.4901	14.3000				68.3001	125.2430

**FIGURE 6:**
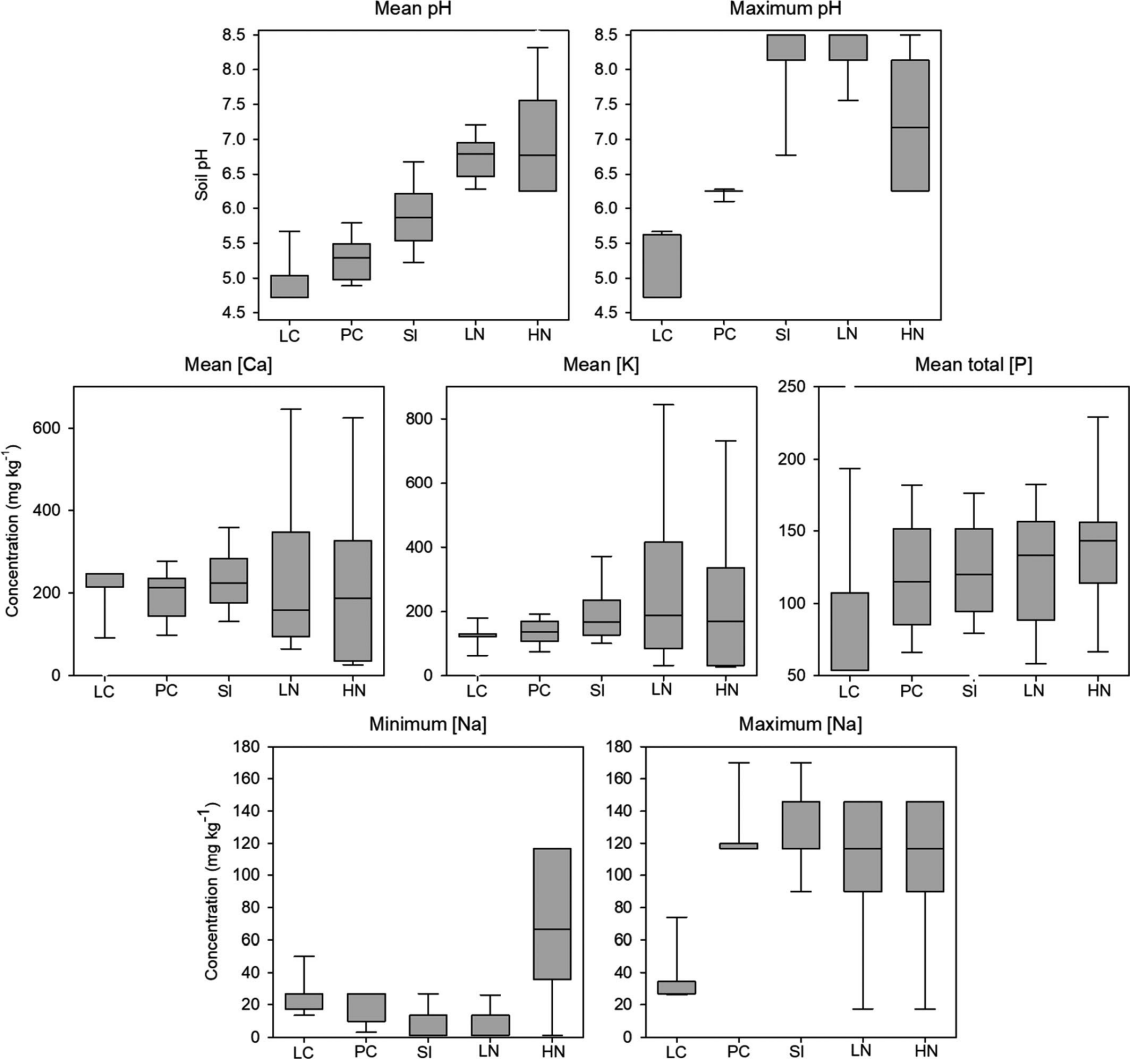
Box plots indicating variability in significant soil parameters (mean and maximum soil pH, mean soil calcium [Ca], potassium [K], and total phosphorus [P], and minimum and maximum soil sodium [Na]) among classified calcifuge plant strategy groups for 538 species from the Mid‐West region of Western Australia. LC: *likely calcifuge*. PC: *possibly calcifuge*. SI: *soil indifferent*. LN: *Likely not calcifuge*. HN: *highly likely not calcifuge*

**FIGURE 7:**
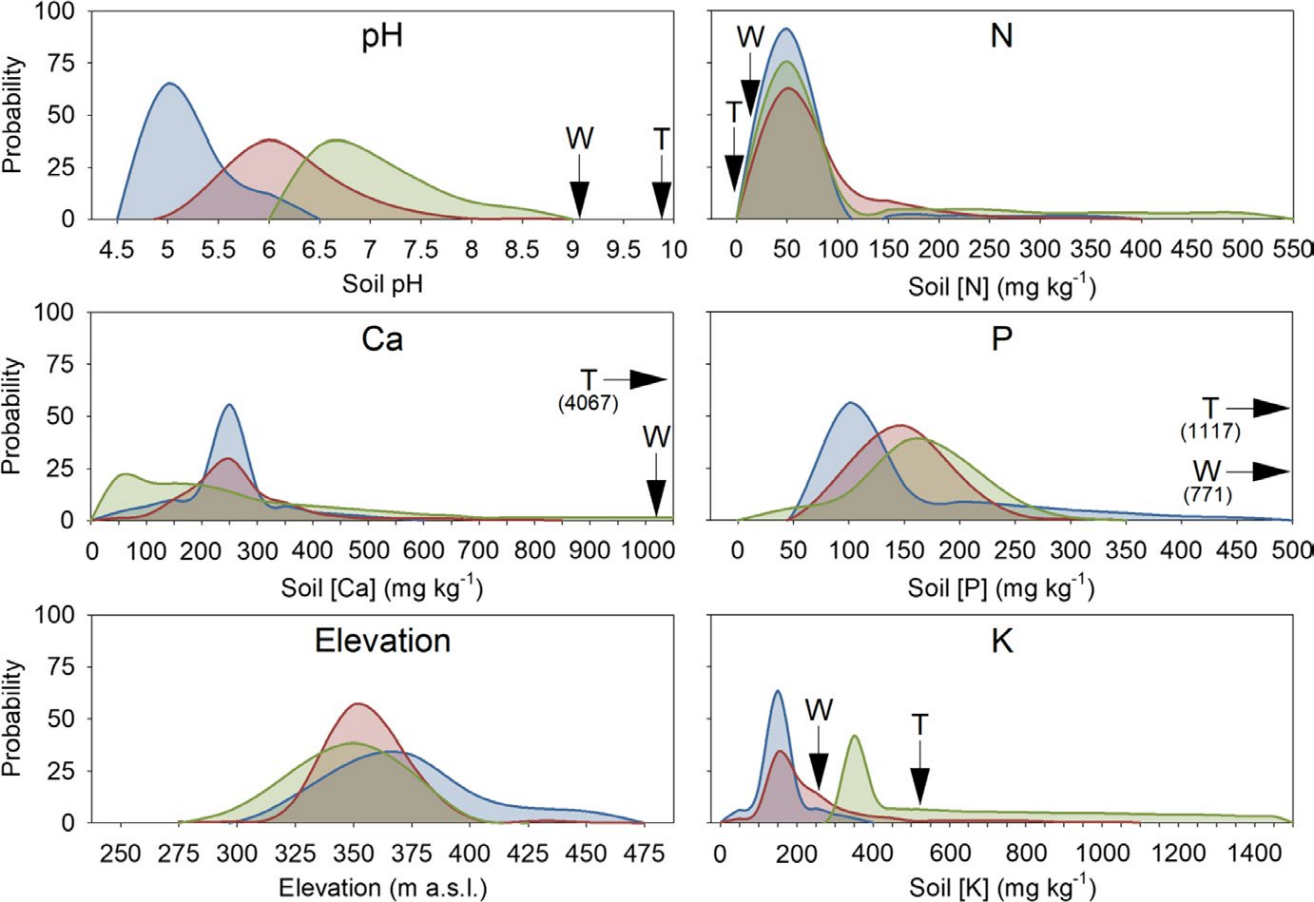
Probability of occurrence curves for species likely to be calcifuge (those classified as *likely calcifuge* and *possibly calcifuge*; blue), species classified as *soil indifferent* (red), and species unlikely to be calcifuge (those classified as *likely not calcifuge* and *highly likely not calcifuge*; green) by selected soil parameters (mean soil pH, mean calcium [Ca], mean elevation, and mean total nitrogen [N], phosphorus [P], and potassium [K]) for 538 species from the Mid‐West region of Western Australia. Arrows indicate comparative characteristics (with exact values in parentheses) for waste rock (W) and tailings (T) produced from mining operations at the study site (from Cross et al., 2018)

Species that were *highly likely not calcifuge* always exhibited significantly higher values of mean soil [K] and total [P] than species that were *likely calcifuge*, and species that were *highly likely not calcifuge* and *likely not calcifuge* exhibited markedly greater range in soil [Ca], [K], and [Na] compared with species that were *likely calcifuge* and *possibly calcifuge* (Figure 6). Probability of occurrence curves indicated clear differences in soil parameter ranges for mean soil pH, [Ca], elevation, total [N], total [P], and [K] among classified calcifuge plant strategy groups (Figure 7).

BEST analyses indicated the most parsimonious environmental variable selection explaining the observed patterns of calcifuge plant strategy proportions included landscape position and soil [Ca] (Pearson *R* = 0.082). ANOSIM indicated dissimilarity in calcifuge plant strategy proportions among landscape position (*R* = 0.005, *p* = .001), with the composition of crests and ridges being markedly more dissimilar from both plains (*R* = 0.223, *p* = .001) and slopes (*R* = 0.128) than these two landscape positions were from one another (*R* = 0.010, *p* = .003). At least 75% of the dissimilarity among landscape position was explained in all cases by variation in the proportional abundance of *soil indifferent* (50%–52% contribution) and *likely calcifuge* (14%–26% contribution) species. The most parsimonious DistLM explained 12.1% of variation in the proportion of calcifuge plant strategies (Table 3) and included landscape position, EC, [K], [Fe], total [P], [Na], elevation, and total [N] as significant predictors (Figure 4b).

### 3.4 Patterns and predictors of nutrient‐acquisition strategies

The most common nutrient‐acquisition strategies included AM (305 species, 56.6%), NM (84 species, 15.6%), NF (66 species, 12.3%), and AM/ECM (38 species, 7.1%), with less representation of CR (21 species, 3.9%), other highly specialized strategies such as carnivorous plants, orchids, and holo‐ and hemiparasites (20 species, 3.8%) and AM/ECM/NF/CR (5 species, 0.9%). One‐way MANOVA indicated there was a significant difference between landscape position in the proportion of nutrient‐acquisition strategies, *F*(14, 1644) = 9.375, *p* < .001, Pillai's Trace = 0.148, partial *η* ^2^ = 0.074. Relevés on plains contained significantly greater proportions of NM, AM/ECM, and NF species than relevés on crests and ridgelines, usually with intermediate values for relevés on slopes (Figure 5b). Relevés on crests and ridgelines exhibited the greatest proportions of AM species, while AM/ECM/NF/CR species were proportionally most abundant on slopes. No patterns were evident among landscape position for the occurrence of CR or other strategies. There was no significant difference between classified calcifuge strategies on the proportion of nutrient‐acquisition strategies, *F*(20, 2,132) = 1.125, *p* = .315, Pillai's Trace = 0.042, partial *η* ^2^ = 0.010.

The most parsimonious multinomial logistic regression models predicting nutrient‐acquisition strategy included mean soil pH, [K], [P], and [Fe], minimum [N] and [P], and maximum EC, [Ca], [K], [Na] and [P] as significant parameters (Table 5). The occurrence of NM species was predicted by increasing soil [Fe], and AM/ECM species were positively predicted by increasing soil total [N] and rarely occurred on the most N‐impoverished soils (mean minimum total [N] 11.7 ± 6.3 mg/kg). Nitrogen‐fixing species were most abundant on N‐impoverished and acidic soils (mean minimum total [N] 6.5 ± 0.50 mg/kg, mean soil pH 5.4 ± 0.07), were less likely to occur on soils with high [Na], and generally avoided soils with higher total [P] (mean total [P] 109 ± 6.3 mg/kg, mean minimum and maximum total [P] 37 ± 4.8 and 218 ± 13.3 mg/kg, respectively). The occurrence of CR species was negatively predicted by maximum soil EC, and CR species exhibited the highest mean minimum soil total [P] of any nutrient‐acquisition strategy (77 ± 19.5 mg/kg, double that of all groups, except NM at 54 ± 7.4 mg/kg). Species with other nutrient‐acquisition strategies exclusively occurred on the most N‐impoverished soils (mean minimum total [N] 3.5 ± 0.06 mg/kg).

**TABLE 5:** Parameter estimates from the most parsimonious multinomial logistic regression model fitted to test the main effects of species fixed factors (mean, maximum, and minimum values of pH, EC, [N], [P], [K], [Fe], [Ca], [Na], and elevation, as well as life history, nutrient‐acquisition strategy, and plant family) on nutrient‐acquisition strategy for 538 species from the Mid‐West region of Western Australia

Nutrient‐acquisition strategy	Significant parameters	B	Std. error	Wald	*df*	*p*	Exp(B)	95% confidence interval (Exp(B))	
								Lower	Upper
NM	*Average [Fe]*	0.009	0.004	3.843	1	.05	1.009	1.000	1.018
AM/ECM	*Maximum [Na]*	−0.015	0.007	4.732	1	.03	0.985	0.971	0.998
	*Minimum total [N]*	0.013	0.005	5.473	1	.019	1.013	1.002	1.024
NF	*Mean pH*	−1.162	0.398	8.509	1	.004	0.313	0.143	0.683
	*Maximum [Na]*	−0.012	0.005	5.002	1	.025	0.988	0.978	0.999
	*Minimum total [N]*	−0.146	0.069	4.499	1	.034	0.864	0.755	0.989
	*Mean total [P]*	0.017	0.006	7.451	1	.006	1.017	1.005	1.029
	*Minimum total [P]*	−0.017	0.005	11.644	1	.001	0.983	0.973	0.993
	*Maximum total [P]*	−0.006	0.003	4.366	1	.037	0.994	0.988	1.000
AM/ECM/NF/CR	None								
CR	*Maximum EC*	−0.017	0.008	4.755	1	.029	0.983	0.968	0.998
	*Minimum total [P]*	0.017	0.008	4.097	1	.043	1.017	1.001	1.034
Other	*Minimum total [N]*	−1.656	0.752	4.853	1	.028	0.191	0.044	0.833

Note

AM was used as the reference category.

BEST analyses indicated the most parsimonious environmental variable selection explaining the observed patterns of nutrient‐acquisition strategy proportions included soil pH and [Fe] (Pearson *R* = 0.097). ANOSIM indicated very little dissimilarity in nutrient‐acquisition strategy proportions among landscape position (*R* = 0.000, *p* = .489). The most parsimonious DistLM explained 14.1% of variation in the proportion of nutrient‐acquisition strategies (Table 3) and included landscape position, elevation, soil pH, [Ca], [Fe], [K], total [P], EC, and total [N] as significant predictors (Figure 4c).

## 4 DISCUSSION

### 4.1 Patterns and predictors of soil chemistry

Many of the edaphic variables we examined in this study exhibited strong elevational gradients (Table 1), and landscape position was the strongest predictor of landscape soil chemistry patterns in both univariate (Table 2) and multivariate (Figure 4) analyses. Variation in soil chemistry among landscape positions likely reflects the transport of weathered materials over long geological time scales, as primary minerals present in bedrock are broken down and either leached or retained as secondary minerals during weathering (Payne et al., 1998). The BIF ridges of the study area lie within the Youanmi Terrane of the Archaean Yilgarn Craton, and are Precambrian in origin with an estimated emplacement age of 2.6–3 Ga (Cassidy et al., 2006; Klein, 2005; Myers, 1993). The Yilgarn Craton underwent deep weathering during the Tertiary, particularly in the late Miocene, but there is little evidence for significant weathering since the Tertiary, and the present landscape has likely experienced minimal modification in the last 2.5 My (Wyrwoll, 1988). Low‐lying undulating plains comprise Quaternary deposits of alluvial sands and lacustrine clay of variable depth (Payne et al., 1998), and were generally slightly acidic or alkaline (Figure 2a). In contrast, crests and ridges are predominantly outcroppings of exposed Archaean banded ironstones covered by a thin veneer of colluvial rocky sands (Payne et al., 1998), and these skeletal, acidic soils are chemically dissimilar (particularly pH, [Fe], total soil [P], and [Ca]) to those of the surrounding lower‐relief landscape (Table 1).

Pedogenesis is associated with chemical and biological transformations (Turner & Laliberté, 2015; Turner et al., 2018), and soils generally become more acidic and nutrient‐impoverished over longer periods of weathering and development (Smeck, 1973; Walker & Syers, 1976). For example, while Fe^3+^ oxides are stable and weather slowly, Fe becomes mobile under anaerobic conditions through reduction (Clément et al., 2005) and is taken up readily by roots and transported to aboveground biomass (along with Al and Mn; Lambers & Oliveira, 2019). Fe is returned to the soil surface via leaf litter deposition and can be redistributed over considerable distances through erosion and water movement (Payne et al., 1998). Similarly, P is lost through erosion, runoff, and chemical and biological transformations converting primary mineral phosphate into occluded forms during weathering and pedogenesis (Filippelli, 2002; Laliberté et al., 2015; Turner et al., 2018; Walker & Syers, 1976). This results in highly weathered regolith in ancient and climatically buffered landscapes such as southwestern Australia being typically P‐impoverished (Hopper et al., 2016; Kooyman et al., 2017). The mobilization and redistribution of total [P] and [Fe] through soil weathering probably explains the strong elevational gradient for these minerals in our study site (Table 1), with these elements eroding from crests and ridges and accumulating in areas lower in the landscape.

Higher [Ca] in the soils of crests and ridges compared with plains and slopes likely reflects the presence of nodular or pisolitic (pedogenic) calcretes in the thin gravelly soil overlying freshly weathering bedrock (Payne et al., 1998; Anand & Paine, 2002). Pedogenic calcrete is common in topographically higher areas on ultramafic greenstones such as BIF on the Yilgarn Craton (Payne et al., 1998; Anand & Paine, 2002). It typically comprises small carbonate‐cemented particles that can accumulate in even noncalcareous regolith overlying mildly calcareous or noncalcareous parent bedrock (Arakel, 1982; Dan, 1977; Read, 1974). Nodular calcretes on ultramafic bedrock are generally present as dolomite, rather than the calcite‐dominated Ca‐rich accumulations more commonly present lower in the landscape (Anand & Paine, 2002), and may be biologically induced through interaction with plant roots and the metabolites of soil microbes (Alonso‐Zarza & Wright, 2010). Dolomite is less soluble and reactive than calcite (Lund et al., 1973, 1975), and consequently [Ca] in the soils of BIF crests and ridges was half to one‐third less than has previously been reported for calcite‐dominated calcrete formations in the region (Anand & Paine, 2002). Similar trends of [Ca] increase along altitudinal gradients have been observed for gneissic‐derived soils with ironstone fragments in southwest Sri Lankan tropical forest, where bedrock geology transitioned from silaceous at low elevation to gneissic at mid‐elevation, and became rich in Ca‐containing feldspars at high elevation (Ediriweera et al., 2008). As such, although calcareous Ca‐rich soils are patchily distributed in areas of low relief across the study site (Payne et al., 1998), the skeletal soils of BIF crests and ridges are likely noncalcareous and contain Ca in forms that are poorly available to plants.

### 4.2 Patterns and predictors of floristic species richness

Although sampling covered only 0.04% of the study area (a total of 33.2 ha), relevés included representation of every soil system and landscape feature, and species accumulation curves indicated that sampling likely captured regional floristic diversity adequately (Figure 3a). The study site exhibited high floristic biodiversity, with 538 plant species recorded from the 830 relevés analyzed and species richness ranging from 2 to 72 per 400 m^2^ relevé. Both total floristic diversity and the most species‐rich genera (particularly *Acacia, Eremophila, Eucalyptus, Grevillea* and *Rhodanthe*) were similar to those previously reported for BIF in the Yilgarn, although the species richness of relevés on crests and ridges in our study site (9–64 species) was slightly higher than values reported for other Yilgarn BIF (5–49 species; Gibson et al., 2010). This greater diversity likely reflects the more transitional location of our study site on the boundary between the extra‐dry and semidesert Mediterranean regions, compared with the more semidesert Mediterranean region situation of previously studied BIF.

Our data support previous observations that the vegetation communities of weathered ironstone outcrops are typically more species‐rich than the surrounding vegetation matrix (Gibson et al., 2010). Approximately 7% of regional floristic diversity (38 species) was recorded only from the slopes and/or crests and ridges of BIF in our study area. Floristic species richness was most strongly predicted by landscape position and increased markedly along an elevational gradient such that vegetation on crests and ridges was, on average, twofold more diverse than the vegetation of plains (Table 1). However, landscape position appeared to be a strong proxy for edaphic factors including soil pH and total [P], and there was a strong negative correlation between floristic diversity and total [P] (Table 2). Both species richness and functional diversity are closely linked to soil infertility in ancient, highly weathered landscapes such as the Mid‐West, and P‐impoverished soils typically harbor the richest and most functionally diverse plant communities (Zemunik et al., 2015, 2016). Available [P] amounts to approximately 5% of total [P] in soils averaged across the Southwest Australian Botanical Region (SWAFR) and is generally between 0.5 and 6 mg/kg in geologically ancient SWAFR soils (Lambers et al., 2010). Most [P] in BIF soils is retained in apatite and is predominantly unavailable to plants, with available [P] representing only approximately 2.0%–2.5% of total [P] (Cross et al., 2019; Cross & Lambers, 2017; Kumaresan et al., 2017). Thus, P is poorly available in the soil of BIF crests and ridges in the study site (mean available [P] 1.4 mg/kg, ranging from 0.1 to 6.8 mg/kg) compared with the more fertile soils of surrounding undulating plains. Phosphorus impoverishment likely represents a major driver of floristic biodiversity in the Mid‐West.

Topography and soil chemistry have been proposed as major drivers of plant distribution in the Mid‐West, and previous studies have suggested that the vegetation of BIF ridges exhibits higher rates of edaphic endemism and is both structurally and compositionally different from the vegetation of the surrounding, predominantly low relief, landscape (Markey and Dillon, 2008; Gibson et al., 2010). Our study provides strong support for an interlinked role of topography and soil chemistry as primary filters to vegetation assemblages in the region. However, both Markey and Dillon (2008) and Gibson et al. (2010) examined vegetation diversity and turnover within and among BIF crests and ridges, and did not explicitly analyze dissimilarity between BIF ridges and surrounding landscape matrices. While the vegetation of relevés from BIF crests and ridges examined in this study was compositionally different from surrounding vegetation from communities on the undulating plains, analyses yielded little evidence of clear spatial patterns in species composition across the study site (Figure 2b). Instead, high rates of species turnover were ubiquitous across the landscape, and the majority of compositional dissimilarity among landscape position (50%–70%) was driven predominantly by variation in the abundance of a few regionally abundant species. This suggests that vegetation communities in the study site (predominantly *Acacia*‐dominated shrubland) are defined by species dominance, rather than incidence, in contrast to the incidence‐defined patterns reported for sandplain (diverse sclerophyllous heathland and low shrubland) and mallee (woodlands dominated by *Eucalyptus* that are multibranched at ground level) vegetation in adjacent regions of southwest Western Australia (Gibson et al., 2017).

While our data provide support for the strong role of topography and soil chemistry as drivers of species occurrence and plant community composition in the Mid‐West, we suggest that high rates of species turnover at fine scales may be typical of the regional Mid‐West flora broadly, rather than being a unique characteristic of BIF habitats. High rates of species replacement are well documented for the SWAFR (e.g., Hopper & Gioia, 2004; Jones et al., 2016; Sander & Wardell‐Johnson, 2011) and from similarly highly weathered and nutrient‐impoverished soils from Brazil (Silva Mota et al. 2018). However, species turnover in Australian arid zone vegetation appears uncorrelated with either climatic or edaphic factors (Gibson et al., 2017), and a large proportion of variation in species composition among relevés across our study site (ca. 88%) was not explained by the most parsimonious DistLM model, supporting a role of other factors contributing strongly to species turnover. High levels of unexplained variation in species composition (ca. 65%) have been reported previously among relevés from several BIF ridges on the Yilgarn Craton (Gibson et al., 2010), and we suggest that future research should attempt to elucidate the primary drivers of species turnover in arid, geologically ancient regions such as the Mid‐West of Western Australian.

### 4.3 Patterns and predictors of plant strategies

Landscape position was the strongest predictor of variation in the functional composition (i.e., the proportion of calcifuge plant strategies and nutrient‐acquisition strategies) of vegetation in the study site, in combination with chemical parameters tightly linked to landscape position such as soil total [P], total [N], soil pH, EC, [Ca], [K], and [Na]. Vegetation assemblages from the crests and ridges of BIF were functionally dissimilar from those of the surrounding landscape, harboring significantly different proportions of species with different calcifuge and nutrient‐acquisition strategies. The majority of species in our study site were *soil indifferent* (62%), occurring over a wide range of edaphic conditions (Figure 5a). A further 26% were *likely calcifuge* or *possibly calcifuge,* while only 12% were *likely not calcifuge* or *highly likely not calcifuge*, likely reflecting most soils in the study site being at least slightly acidic. Landscape position and soil [Ca] were the strongest environmental predictors of observed patterns in calcifuge plant strategy, with *highly likely not calcifuge* and *likely not calcifuge* species exhibiting significantly higher mean values and markedly greater variability in soil [Ca], [K], and total [P] than species that were *likely calcifuge* and *possibly calcifuge* (Figure 6). The vegetation of BIF crests and ridges harbored proportionally more *likely calcifuge* and *possibly calcifuge* species than communities on adjacent slopes and plains, and the majority of species recorded only from higher elevation landscape positions were *likely calcifuge* or *possibly calcifuge*. As such, it appears that the edaphic conditions of BIF crests and ridges select for or at least offer the most suitable regional conditions for the establishment of calcifuge species.

While the classification of calcifuge species can be accurately inferred from their distribution along soil pH gradients (Clarkson 1966; Grime & Lloyd, 1973; Etherington, 1981; Lee, 1999), the classification of calcicole species requires a deeper understanding of plant–soil interaction and soil parameters, particularly soil exchangeable [Ca] (De Silva, 1934; Lee, 1999; White and Broadley 2003; Gao et al., 2020). Early studies examining calcifuge and calcicole plant strategies found calcifuge species to be generally restricted to soils with <6 mmol/dm^3^ soil exchangeable [Ca], while calcicole species typically occurred in soils where soil exchangeable [Ca] was 10–30 mmol/dm^3^ (e.g., De Silva, 1934; Jefferies and Willis, 1964). Chemical analyses indicated that most soils sampled in the present study were low in exchangeable [Ca] (mainly 0.2–5.5 mmol/dm^3^, rarely up to 25 mmol/dm^3^), including samples collected from low‐lying areas identified as harboring calcareous soils (Payne et al., 1998; Anand & Paine, 2002). However, the above exchangeable [Ca] thresholds for calcicole species were derived from studies in European ecosystems and may reflect experience with geologically young landscapes where insufficient time has elapsed for Ca to be leached from soils. It might be expected that comparatively less Ca would be present in the heavily weathered soils of geologically ancient regions, as examination of multi‐million‐year soil chronosequences in southwest Western Australia indicates cation concentrations decline markedly from youngest to oldest chronosequence stages (e.g., Laliberté et al., 2012). However, rather than being excluded from heavily weathered soils where the concentration of exchangeable [Ca] or other important cations may be very low, calcicole species in geologically ancient landscapes likely evolved different strategies to meet their ecological and nutritional requirements. For example, *Callisthene fasciculata* (Vochysiaceae) is an Al‐accumulating calcicole occurring on acidic (pH 5.6) but Ca‐rich soils (57.5 mmol/dm^3^ [Ca]) with apparently no exchangeable [Al] in the Brazilian Cerrado savanna (de Souza et al., 2020), while the calcicole *Grevillea thelemanniana* (Proteaceae) occurs on acidic (pH 3.9–6.7) and Ca‐impoverished soils (mostly <3 mmol/dm^3^ [Ca]) in the Bassendean dunes of southwest Western Australian and accumulates Ca in leaf tissues to balance accumulation of trans‐aconitate (Gao et al., 2020).

The functional diversity of nutritional strategies in plant communities is linked to soil fertility, and P‐impoverished soils generally harbor an abundance of functional diversity as well as overall species richness (Zemunik et al., 2015, 2016). The stoichiometry of soil N and P, in combination with organic carbon, is a significant driver of functional diversity in vegetation assemblages (Sýkora et al., 2004; Wardle et al., 2004; Laliberté et al., 2014). Soils change from N‐limiting to P‐limiting for plant productivity over long periods of weathering and pedogenesis, resulting in shifts in the dominance of nutrient‐acquisition strategies along natural soil chronosequences (Hayes et al., 2014; Zemunik et al., 2016; Teste et al., 2016). Vegetation on the oldest and most P‐impoverished soils in ancient landscapes is typically characterized by species with root traits that enhance P acquisition based on the release of carboxylates such as specialized cluster, capillaroid, dauciform and sand‐binding roots (Laliberté et al., 2015; Lambers et al., 2018). Mycorrhizal symbionts allow for exploration of greater soil volumes in the acquisition of P and other poorly mobile nutrients (Lambers et al., 2018), but are ineffective when P availability is very low (Lambers et al., 2018; Parfitt, 1979). In contrast, the vegetation of geologically younger, less developed and N‐impoverished soils is typified by greater abundance of N_2_‐fixing species and species with no mycorrhizal P‐acquisition strategy (Lambers et al., 2018; Lambers & Oliveira, 2019; Zemunik et al., 2015, 2016). Although the presence of CR and NM species was relatively constant among landscape positions in our study site (Figure 5b), AM species became markedly more abundant with increasing elevation and predominated on BIF crests and ridges, while the abundance of NF species decreased along the same landscape position gradient. However, being mycorrhizal does not necessarily evidence that the plants relied on mycorrhizal fungi for their P acquisition (Lambers et al., 2018; Teste et al., 2016).

Although elevation was among the significant predictors of species richness, species composition, and the proportion of calcifuge plant strategies and nutrient‐acquisition strategies in both univariate and multivariate analyses, we assume elevation is likely a proxy for BIF in the Mid‐West as previously proposed by Tomlinson et al. (2020). The crests and ridges of BIF in the study site are <200 m higher in the landscape than surrounding topography, and elevational gradients in edaphic and vegetative parameters probably reflect the increasing incidence of the shallow, well‐drained soil characteristic of BIF (Gibson et al., 2010), rather than altitudinal climatic shifts (Tomlinson et al., 2020). The availability of soil moisture in the upper soil profile (200–1,000 mm) has been identified as a critical factor governing the occurrence of edaphic endemics on BIF (Tomlinson et al., 2020), and at least some BIF species likely rely upon water stored in cracks and fissures in bedrock, particularly during early seedling establishment (e.g., Poot & Lambers, 2003; Robinson et al., 2019; Tomlinson et al., 2020). BIF are likely water‐gaining landforms (Payne et al., 1998), and as topographically complex features in an otherwise relatively uniform arid environment landscape matrix, they likely represented comparatively mesic refugia during periods of increased aridity (Byrne et al., 2017). The topographic complexity of BIF is likely a stronger contributor to being a refugium than their elevation (Byrne et al., 2017) and probably offers a variety of ecological niches, facilitating the stochastic establishment of both ecological specialists and generalists in an otherwise relatively homogenous landscape matrix. The incorporation of rock fragments in soils, for example, markedly alters soil thermal and hydrological dynamics and improves the range of niches available for seed germination and establishment, even at small scales (e.g., Bailey et al., 2012; Cross & Lambers, 2017; Masarei et al., 2020; Renison et al., 2005). Although further empirical study is required to elucidate the seasonal and long‐term hydrological dynamics of BIF soils, the expansion and contraction of species from such refugia over long periods of climatic fluctuation would explain why BIF harbor such a high proportion of regional biodiversity, rather than compositionally unique floristic assemblages. Our results support the hypothesis of Gibson et al. (2010) that Yilgarn BIF ranges are palimpsests (i.e., an overlay of different assemblages that have arisen and been fully or partially erased over a long time), their contemporary vegetation communities likely reflecting a long and complex history of multiple colonizations, extirpations, recolonizations, and isolation.

Edaphic endemics on BIF are range‐restricted species exhibiting traits associated with specific nutritional, recruitment, or physiological requirements, commonly including specialized drought‐avoidance or drought‐tolerance strategies (Poot & Lambers, 2003; Nunes et al., 2015; Tomlinson et al., 2020; Elliott et al., 2019; Rajapakshe et al., 2020). These traits may be genetically fixed and may be selected against or place species at a competitive disadvantage on the deeper soils typical of areas lower in the landscape (Poot & Lambers, 2003). Many of the species recorded from BIF in our study site were broadly distributed and occurred along edaphic gradients. Over half the regional flora were represented in relevés from BIF crests and ridges, and only a small suite of species (about 7% of total biodiversity) were recorded only from crests and ridges (13 species) or from slopes and crests and ridges (24 species). While some of these species appear to be restricted to rock outcrops and skeletal soils over rock (e.g., *Eucalyptus petraea, Isotoma petraea, Dodonaea adenophora, Micromyrtus sulphurea* and *Tetragonia eremaea*), the majority are widespread in the Mid‐West region (FloraBase 2020). Only one species in the study site, *Acacia woodmaniorum,* appears to be an edaphic endemic, recorded only from BIF in the Yilgarn Craton (Markey and Dillon, 2008; Millar et al., 2013). Rather than BIF vegetation in our study site harboring high proportions of edaphic endemics that cannot establish or are inferior competitors in the surrounding landscape (Poot & Lambers, 2003), we surmise that plant communities on BIF instead represent musea of regional floristic biodiversity. The only species not represented on these landforms are those that cannot establish or are inferior competitors in heavily weathered, acidic, skeletal, and nutrient‐impoverished soils.

### 4.4 Implications for rehabilitation and ecological restoration

Mining operations in Western Australia, as in many other jurisdictions around the world, are typically expected or required to re‐establish representative, functionally diverse locally native vegetation communities following disturbance (Cross et al., 2017; Stevens & Dixon, 2017). Studies have suggested this will be challenging on highly altered substrates such as waste rock or alkaline mine tailings, because the chemical characteristics of processed mined materials differ starkly from those of natural soils (Cross & Lambers, 2017; Cross et al., 2017). Landforms requiring postmining ecological restoration in the Mid‐West region generally comprise waste rock or tailings material overlain by a shallow (ca. 300 mm) surface cover of natural topsoil and waste rock (Cross et al., 2018, 2019; Kumaresan et al., 2017). Our data indicate that that these mined materials present a chemical environment quite unlike anything that native flora likely encounter throughout their natural distribution (Figure 7).

Plant‐growth studies showed that high pH and insufficiently available N are likely primary edaphic constraints to vegetation establishment on alkaline postmining landforms (Cross et al., 2019, 2020), and it was proposed that the edaphic conditions of these materials may select against high proportions of biodiversity where the vegetation to be reinstated originates from heavily weathered acidic soils (Cross et al., 2019). Our data support high pH and insufficiently available N as likely filters to vegetation establishment in the study site, but suggest that soil [Ca], total [P], and possibly total [K], may also represent key filters. Previously published values of pH for waste rock produced in the Mid‐West range from 8.0 to 9.5 (e.g., Commander et al., 2020; Cross et al., 2018, 2019; Golos et al., 2019). These values lie significantly outside the range we report for species likely to be calcifuge in the region (pH 4.5–6.5) and at the upper limit of the range reported for species recognized as either soil indifferent or unlikely to be calcifuge. The pH reported for magnetite tailings (>9.5) markedly exceeds that of any natural soil sampled in the study site. Natural soils in the Mid‐West contain high total [N] (typically 30–70 mg/kg), while both waste rock and alkaline tailings are strongly or entirely N‐depleted (Cross et al., 2018, 2019). Values of soil total [P] and [Ca] previously reported for both waste rock (600–900 mg/kg and 1,200–1,600 mg/kg, respectively) and magnetite tailings (600–900 mg/kg and 4,000–4,900 mg/kg, respectively) greatly exceed the concentration of total [P] and [Ca] we report for Mid‐West soils (typically ≤500 mg/kg and ≤1,000 mg/kg, respectively).

Based on results from logistic regression models (Table 5), we suggest that the edaphic environment of waste rock and magnetite tailings will likely select strongly against AM/ECM species (the frequency of AM/ECM species was positively correlated with total soil [N], while these substrates are typically N‐depleted) and NF species (the frequency of NF species was negatively correlated with total soil [P], while these substrates typically have very high total [P]), together representing approximately 20% of the regional species pool (104 species). Selection against AM/ECM species on N‐impoverished soils may stem from poor establishment potential from seed, as the seeds produced by most AM/ECM species in the Mid‐West are very small (<1 mm) and possess minimal [N] in seed tissues (Cross et al., 2019; A.T. Cross unpubl.). While NF species occurred with greater frequency on N‐impoverished soils, they were less common on alkaline soils and soils with higher total [P] and [Na]; the performance of NF species on postmining substrates may be idiosyncratic, as at least some species from the study site appear to establish and develop comparably on magnetite tailings and natural topsoils (Cross et al., 2019). Calcifuge species (representing approximately a quarter of regional biodiversity) will also likely be strongly selected against or excluded from waste rock and tailings landforms, as these species solubilize P and micronutrients poorly at high pH (Zohlen & Tyler, 2000, 2004) and can exhibit compromised root development when rhizosphere Ca concentrations exceed 1 mM (Jefferies and Willis, 1964; Lee & Woolhouse, 1969; Burstrom, 1968). Phosphorus‐sensitive plants (Lambers & Oliveira, 2019) may also be excluded (ca. 4% of species in the study area), as CR species were predicted by increasing total soil [P], but the very high total [P] in waste rock and magnetite tailings may result in potentially toxic levels of biologically available soil P (Cross & Lambers, 2017).

It is probable that NM and AM species (together comprising 72% of species in the study area), particularly NM species (16% of species in the study area), are least likely to be selected against on heavily altered substrates. NM species (excluding CR species) possess no specific strategies for nutrient‐acquisition (Lambers & Oliveira, 2019), and recent research has found NM *Maireana* species from the Mid‐West are successful pioneers on alkaline tailings due to effective scavenging of trace amounts of N from the substrate and exceptionally high leaf N‐resorption efficiency from senescent leaves (Zhong et al., 2021). While the lack of available N in substrates such as waste rock and magnetite tailings might appear to present conditions selecting for species with other nutrient‐acquisition strategies (e.g., holo‐ and hemiparasites, carnivorous plants, and orchids; 4% of species in the study area) as these species typically inhabit N‐impoverished soils, it is highly unlikely that restored postmining landforms offer the highly specialized ecological niches and biological mutualisms that such species typically require (e.g., Gravendeel et al., 2004; Těšitel, 2016; Clarke et al., 2018).

## 5 CONCLUSIONS

BIF are topographically and edaphically complex landforms of great geological antiquity and high biodiversity value. Their topographic prominence and complexity in an otherwise relatively homogenous landscape has likely facilitated the accumulation of many species occupying diverse ecological niches over a long time, with increasing P limitation promoting greater plant diversity and functional diversity along an elevational gradient from lower slopes to crests and ridgelines. As such, we propose that BIF represent important musea of regional floristic biodiversity, being comparatively mesic refugia providing conditions suitable for the persistence of entire regional species pools, except species that cannot establish on, or are inferior competitors in, heavily weathered, acidic, skeletal, and nutrient‐impoverished soils. Vegetation assemblages of BIF crests and ridgelines represent concentrated hotspots of plant species diversity rather than harboring vegetation assemblages that are compositionally unique from the matrix of vegetation assemblages occupying the surrounding landscape. However, they harbor a unique suite of edaphic endemics including the majority of regional species exhibiting calcifuge plant strategies and should be considered of extremely high conservation value. It is highly improbable, if not completely implausible, that landforms representative of the topographic and edaphic complexity of undisturbed BIF could be artificially recreated.

As the edaphic conditions of postmining landforms (i.e., waste rock dumps, tailings storage facilities) in the Mid‐West typically differ starkly from the predisturbance landscape of undisturbed BIF, the establishment of native vegetation communities that are compositionally and functionally representative of those on undisturbed BIF on postmining landforms will likely be extremely challenging. To avoid rehabilitation or ecological restoration failure on highly altered substrates such as those produced by mining, we must consider the edaphic environment (physical, chemical, hydrological, and biological) offered by the substrate and how this differs from the soils in which desired vegetation communities naturally occur. Where the characteristics of these materials are markedly divergent, for example, in key parameters such as pH, [Ca], or total [N], [P], or [K], significant investment may be required to i) determine the impact of divergent parameters on species intended for return to disturbed lands or reconstructed landforms, to identify which species or functional groups may be selected against or excluded from these areas; ii) identify organisms with traits, life histories or distributions conferring some level of tolerance to the expected edaphic conditions, and determine the potential of these species to facilitate or accelerate ecosystem processes such as soil development; and iii) explore options to ameliorate the edaphic environment and more closely align it with the conditions presented by natural substrates (e.g., through acidification and improving N availability; Cross et al., 2019; Cross & Lambers, 2017). As research programs addressing these requirements may take years to develop, we propose that practitioners such as the mining industry must consider the edaphic environment of landforms requiring rehabilitation or ecological restoration, and its implications for vegetation establishment and plant community development, at the very earliest stages of planning or environmental impact assessment.

## CONFLICT OF INTEREST

None declared.

## AUTHOR CONTRIBUTIONS

**Adam T. Cross:** Conceptualization (equal); data curation (lead); formal analysis (lead); funding acquisition (lead); investigation (equal); methodology (lead); project administration (lead); resources (lead); software (lead); supervision (equal); validation (lead); visualization (lead); writing‐original draft (lead); writing–review and editing (equal). **Hans Lambers:** Conceptualization (equal); investigation (equal); supervision (equal); writing–review and editing (equal).

## Supporting information

Supplementary Material

## Data Availability

Data are unable to be publicly archived due to legal requirements, but are available from the corresponding author upon request.
