# Supplementary material for: Calcicole–calcifuge plant strategies limit restoration potential in a regional semi‐arid flora
Source: Ecol Evol. 2021 May 1;11(11):6941–61. doi: 10.1002/ece3.7544 (PMC8207153; doi:10.1002/ece3.7544)
Supplement: Supplementary file 1 — Supplementary Material [file ECE3-11-6941-s001.docx]

**Appendices**


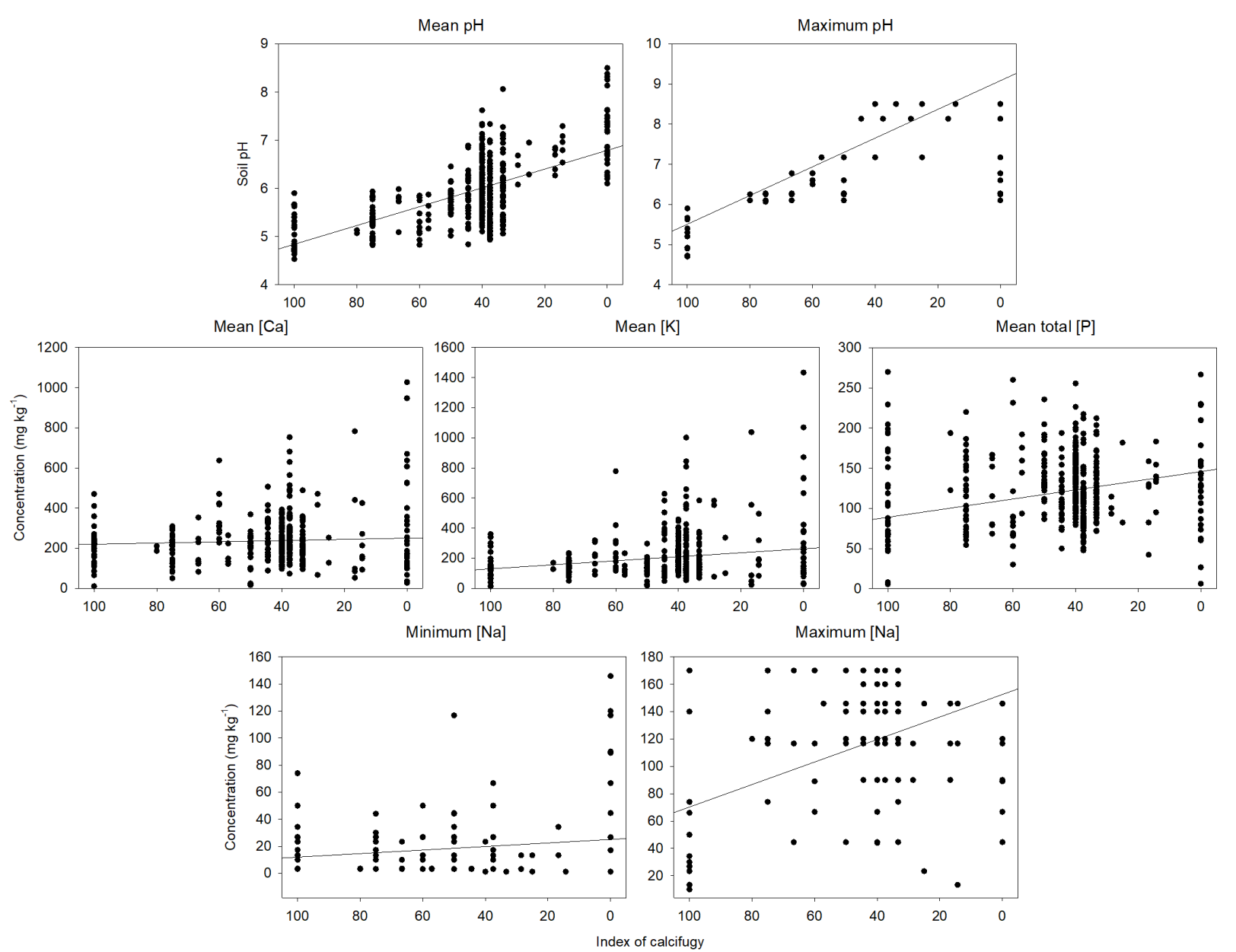


**Figure S1**. Scatter plots with fitted regression lines indicating relationship between mean, minimum and maximum soil pH and index of calcifugy (*IC*, top), mean soil [Ca], [K] and [N] and *IC* (middle), and mean soil [P], [Na] and minimum [Na] and *IC* (bottom) for 538 species from the Mid-West region of Western Australia.

**Table S1**. Parameter estimates from the most parsimonious multinomial logistic regression model fitted to test the main effects of species fixed factors (mean, maximum and minimum values of pH, EC, N, P, K, Fe, Ca, Na and elevation, as well as life history, nutrient-acquisition strategy and plant family) on nutrient-acquisition strategy for 538 species from the Mid-West region of Western Australia. AM was used as the reference category.

| **Nutrient-acquisition Strategy** | **Significant parameters** | **B** | **Std. Error** | **Wald** | **df** | ***P*** | **Exp(B)** | **95% Confidence Interval (Exp(B))** | |
| --- | --- | --- | --- | --- | --- | --- | --- | --- | --- |
|  |  |  |  |  |  |  |  | **Lower bound** | **Upper bound** |
| AM | None |  |  |  |  |  |  |  |  |
| AM/ECM | Maximum P | 0.007 | 0.003 | 3.83 | 1 | 0.050 | 1.007 | 1 | 1.014 |
| NF | Mean pH | -1.421 | 0.375 | 14.346 | 1 | <0.001 | 0.241 | 0.116 | 0.504 |
|  | Minimum P | -0.016 | 0.006 | 7.428 | 1 | 0.006 | 0.984 | 0.973 | 0.996 |
|  | Mean P | 0.017 | 0.007 | 5.81 | 1 | 0.016 | 1.017 | 1.003 | 1.032 |
|  | Mean K | 0.003 | 0.001 | 3.912 | 1 | 0.048 | 1.003 | 1 | 1.006 |
| AM/ECM/NF/CR | None |  |  |  |  |  |  |  |  |
| CR | None |  |  |  |  |  |  |  |  |
| Other | Minimum N | -1.656 | 0.752 | 4.853 | 1 | 0.028 | 0.191 | 0.044 | 0.833 |
